# Are changes in pain associated with changes in heart rate variability in patients treated for recurrent or persistent neck pain?

**DOI:** 10.1186/s12891-022-05842-4

**Published:** 2022-10-04

**Authors:** Anders Galaasen Bakken, Andreas Eklund, Anna Warnqvist, Søren O’Neill, David M. Hallman, Iben Axén

**Affiliations:** 1 Department of Environmental Medicine, Unit of Intervention and Implementation Research for Worker Health Karolinska Institutet, Nobels väg 13, S- 171 77 Stockholm, Sweden; 2 Division of Biostatistics, Karolinska Institutet, Nobels väg 13, S- 171 77 Stockholm, Sweden; 3 Spine Centre Southern Denmark, University Hospital of Southern Denmark, Østre Hougvej 55, 5500 Middelfart, Denmark; 4 Department of Occupational Health Sciences and Psychology, University of Gävle SE Centre for Musculoskeletal Research (CBF), Kungsbäcksvägen 47, S-801 76 Gävle, Sweden

**Keywords:** Autonomic nervous system, Heart rate variability, NRS-11, Persistent neck pain

## Abstract

**Background:**

Persistent or recurrent neck pain is associated with perturbations in the autonomic nervous system balance, and nociceptive stimulation has been seen to influence this balance. However, very few prospective studies have addressed the extent to which changes in pain associate with changes in autonomic cardiac regulation. Therefore, we investigated if changes in pain vary with changes in heart rate variability in a cohort of patients treated for persistent or recurrent neck pain.

**Method:**

This analysis is based on data from a randomized controlled trial in which participants were given home stretching exercises with or without spinal manipulative therapy for two weeks. As the effectiveness of the intervention (home stretching exercises and spinal manipulative therapy) was found to be equal to the control (home stretching exercises alone), all 127 participants were studied as one cohort in this analysis. During the intervention, pain levels were recorded using daily text messages, and heart rate variability was measured in the clinics three times over two weeks. Two approaches were used to classify patients based on changes in pain intensity: 1) Clinically important changes in pain were categorized as either "improved" or "not improved" and, 2) Pain development was measured using pain trajectories, constructed in a data driven approach. The association of pain categories and trajectories with changes in heart rate variability indices over time were then analysed using linear mixed models.

**Results:**

Heart rate variability did not differ significantly between improved and not-improved patients, nor were there any associations with the different pain trajectories.

**Conclusions:**

In conclusion, changes in pain after home stretching exercises with or without spinal manipulative therapy over two weeks were not significantly associated with changes in heart rate variability for patients with persistent or recurrent neck pain. Future studies should rely on more frequent measurements of HRV during longer treatment periods.

**Trial registration:**

The trial was registered at ClinicalTrials.gov, registration number: NCT03576846.

**Supplementary Information:**

The online version contains supplementary material available at 10.1186/s12891-022-05842-4.

## Background

Chronic musculoskeletal pain-conditions, including persistent or recurrent neck pain (NP), are associated with altered sympathetic and parasympathetic activity of the autonomic nervous system (ANS) [1–3], commonly measured with heart rate variability (HRV) [4]. HRV is regarded as a biomarker for ANS dysregulation (as an indicator of autonomic cardiac modulation), and a low HRV has been associated with a range of poor health outcomes, such as cardiovascular disease, diabetes, mood disorders, and increased mortality [1, 5].

NP is a common reason to seek care [6] and current guidelines [7, 8] recommend a range of treatment options, including spinal manipulative therapy (SMT), defined as mobilization or manipulation of the spinal joints [9]. SMT alone or in combination with other approaches has been shown to reduce pain in both the short [10, 11] and the long term [12] in patients with NP. Stretching exercises alone or in combination with other treatments are also known to reduce musculoskeletal pain [7, 11, 13].

The mechanisms behind the pain-reducing effect of SMT and stretching are not clear. Thus, there are no objective biomarkers of treatment response. Different mechanisms have been suggested [14, 15], and it has been hypothesized that part of the pain-reducing effects is due to how the treatment influences the ANS balance [14–16]. A recent overview of systematic reviews have suggested that acute, short-term sympathetic upregulation can be observed with SMT [17] which has also been suggested with stretching exercises [18–22]. Further, a pain-reducing effect has been observed in a study of patients with persistent or recurrent NP treated with breathing exercises intended to normalize HRV. As improvements in both HRV *and* pain were observed [23], it suggests that it might be altered ANS balance as indicated by HRV per se that influences the pain, regardless of the type of treatment. In other words, changes in ANS balance following treatment precedes changes in pain perception. However, the proposed mechanism of SMT as having an acute positive effect on the ANS balance is questionable [17], and was challenged in two recent systematic reviews, which concluded that the evidence in favour of such a link was of low or very low quality [24, 25]. Also, a recently published randomized trial investigating acute effects of SMT on HRV using a successful sham treatment found no evidence of such an effect over placebo [26].

The long-term effects of manual therapy on HRV have not been rigorously investigated, but we recently conducted a trial and found no difference in HRV over two weeks between groups receiving SMT and home stretching exercises vs home stretching exercises alone [27].

Considering the fact that experimentally induced pain alters the patients' HRV [28], the mechanism in a clinical setting could perhaps be conceived as working in the opposite direction. In other words, changes in pain are causing the observed HRV changes.

Although our previous research found no significant group difference effect on pain [29] and HRV [27], large variability in both outcomes was found between patients across intervention groups. For example, the root mean squared successive differences (RMSSD) between normal heartbeats (the a primary outcome of HRV) showed both decreases and increases during the intervention period, with confidence intervals ranging from -3.23 ms to 0.28 ms [27], and the proportion of participants reaching a Minimal Clinically Important Difference (MCID) in pain intensity was 46/123 across intervention groups. It was important to explore whether changes in pain varied with changes in HRV in the intervention period as evidence of such an association could shed light on this relationship in a clinical setting and inform the use of HRV as an objective marker of treatment response.

Two strategies were employed to investigate changes in pain during the two-week treatment period. First, patients were categorized based on MCID in pain intensity. Then, patients were classified into detailed pain trajectories in a data driven approach. The two strategies complemented each other; both the actual difference in pain intensity from baseline to the end of the intervention period, and different pain developments throughout the intervention period, were explored.

As noted, an association between reduced HRV and persistent NP has been established. However, it is not known how HRV responds to changes in NP in a clinical setting.

The present study aimed to investigate the association between changes in pain intensity and changes in HRV in patients receiving treatment for NP. We hypothesized that patients experiencing a MCID improvement in pain intensity would show a beneficial increase in HRV over time, and that trajectories with consistent/fast improvement would show a beneficial increase in HRV compared to trajectories that were stable.

## Method

This study is a secondary analysis of data from a Swedish multicentre RCT [30]. The primary aim of the original RCT was to investigate the effect of a series of four treatments over two weeks of SMT plus home stretching exercises vs home stretching exercises alone on pain and HRV for patients with persistent or recurrent NP. The treatment effects on primary outcomes are reported in two previous publications [27, 29]. The study design, recruitment, randomization, and interventions have been described in detail in a published protocol [30] and are reported here in Additional file 1: Appendix A. The trial "The effect of spinal manipulative therapy on HRV and pain in patients with chronic neck pain: a randomized controlled trial " was registered at ClinicalTrials.gov, registration number: NCT03576846.

In the current analysis, we used all participants in the RCT, thus the study group consisted of 127 patients with persistent or recurrent NP residing in the Stockholm area. Participants were recruited through clinic advertisements and newsletters, public postings on the clinics' social media pages, and local newspapers. Patients seeking care at the clinics were also invited to participate. They had experienced persistent or recurrent NP for more than six months and must not have received chiropractic treatment during the previous six months. They had to be minimum 18 years of age and must be able to read and understand Swedish. This represents a relatively small proportion of chiropractic patients, as they more commonly seek care with low back pain and with a shorter duration of pain [31, 32].

Participants were excluded if they reported any of the following conditions: cardiovascular disease, hypertension, cancer, infection, acute cervical radiculopathy, dizziness, previous drop-attacks, or diabetes. They were also excluded if taking any of the following medications: Steroids, β-blockers, or antidepressants. Further, pregnancy, BMI above 30, or recent experience of severe trauma led to exclusion.

**Fig. 1:**
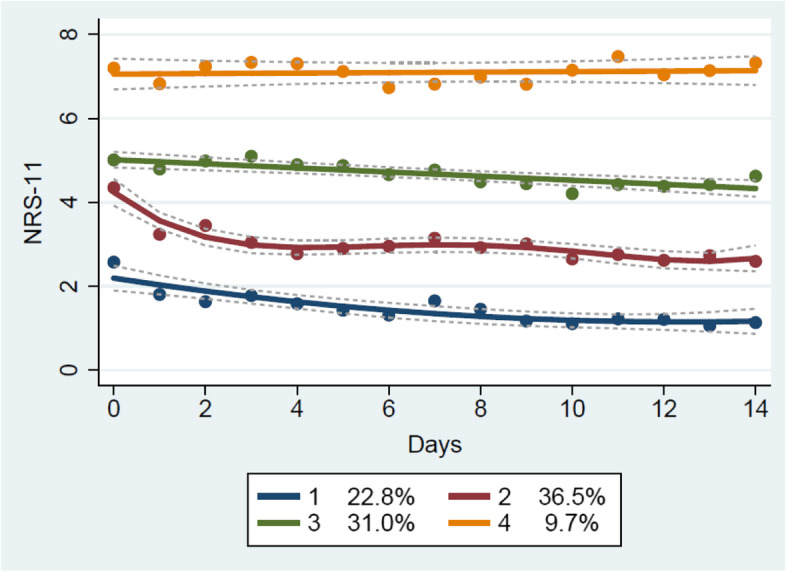
Pain trajectories based on group-based trajectory models [34]. Pain is measured with NRS-11 (0–10) at baseline and each day for two weeks. Numbers in the box show the proportion of study patients belonging to each trajectory

During the two-week intervention, clinical pain was measured daily, and HRV was measured on three separate occasions. Participants were categorised in relation to changes in pain using two strategies:

1. On the basis of MCID in pain intensity, measured with 11-point Numeric Rating Scale (NRS-11), participants were categorized as "improved" (*reduction* ≥ 2/10 from the first measurement to the last) or "not improved" (no change or worsened, change ≤ 0/10)). This approach excluded patients experiencing only small, non-clinically significant clinical improvements from further analysis, ensuring a distinct difference in treatment response between the two categories.
2. Four NP trajectories were identified based on daily text-message reports of pain intensity (NRS-11) during the two-week study period. A latent class analysis was used, and the pain trajectories were estimated with a linear regression model (for trajectories three and four), with a quadratic model (for trajectory one), and a fourth-order model (for trajectory two). The process of choosing the order of the models for the different trajectories was iterative, starting from the simplest (intercept only) model for all trajectories and adding complexity as long as the coefficients remained significant at the 0.05 level. Ordinary regression was chosen as the estimation method, in line with previous analysis [27, 29]. We chose the four-trajectories solution based on AIC (Akaike Information Criteria) and previous research [33]. Also, we achieved relatively detailed trajectories. Models with two or three trajectories were also explored but found to have no advantages over the four-trajectories model. The trajectories were labelled 1–4, based on their pain intensity. Trajectory 1 was chosen as the reference category as it represented the trajectory with the lowest level of pain and was chosen before trajectory 4 (highest level of pain) as it had a higher number of patients (Fig. 1).

### Variables

The participants answered questionnaires covering the suggested domains in the International Classification of Functioning, Disability, and Health (ICF) [35] by measuring impairments, function (limitations), and restrictions to participating in activities. The questionnaires utilized were the Neck Disability Index (NDI) [36], the McGill Questionnaire [37, 38], and the Euroqol-5 dimensions (EQ-5D). In addition, the StarTBack tool [39] and attitudes towards participating were used as a baseline measurement.

### Independent variable

Pain intensity was measured as a self-reported NRS-11 value, ranging from 0 ('No pain') to 10 ('Worst pain imaginable'). NRS-11 is a validated measure of pain intensity [40, 41] and was chosen due to the observed association between pain and HRV in previous studies [1, 2]. Pain intensity was measured at baseline and each day for two weeks (the intervention period) using daily text messages [42].

### Dependent variable

HRV is a measure of the variability in time latency between heartbeats [43]. It is known to quickly adapt to changing circumstances [43] and is recognized as a reliable [44] measure of the function of the ANS, where a high HRV index indicates a well-functioning, responsive ANS and vice versa [1]. HRV was measured at baseline, one week and two weeks during the intervention using FirstBeat, a small, portable device attached to the chest measuring Electrocardiography (ECG). The participants were instructed to avoid caffeine, alcohol, tobacco, and strenuous exercise the same day as the measurements. The measurements were done prior to the interventions, to prevent any direct influence of treatment on HRV. The measurements were performed with the patient seated in a chair, wearing hearing protection, facing the wall, instructed to breath normally during this time. Efforts were also made to keep temperature and lighting in the room at the same level at each measurement. The measurement was undertaken during a normal working day (between the hours of 0700–1600). A five-minute relaxation period was used before HRV was obtained as resting HRV the following five minutes.

Data were extracted from the ECG recordings as time intervals between successive ECG R-waves (R-R intervals). Five minutes segments were used when analysing HRV indices in both time and frequency domains.

The HRV data were cleaned for artifacts and ectopic beats (i.e., common changes in a heartbeat involving an extra or skipped heartbeat) to ensure sufficient quality. The R-R intervals were visually inspected using Kubios software [45]. If the data had insufficient quality, different sensitivity filters, ranging from 0.45 to 0.05 s differing from the local average, were utilized to remove artifacts. If the proportion of excluded artifacts exceeded 5% when the data gained sufficient visual quality, the sample was excluded [46].

The Taskforce of the European Society of Cardiology and the North American Society of Pacing and Electrophysiology [47] has developed standards of measurements of HRV, which were used with adaptations in this study. We did not include Low Frequency (LF) power and the LF/HF ratio as their physiological interpretation is unclear [48–50]. HRV measurements include various indices, measuring different parts of the ANS. These are summarized in Table 1.

**Table 1:** Heart Rate Variability indices suggested by The Taskforce of the European Society of Cardiology and the North American Society of Pacing and Electrophysiology [27]

**HRV indices**	**Indicator of**	**Domain measure**	**Change that improves HRV**
**R-R interval**	Global HRV activity	Time	Increase
**Root mean squared successive differences between IBIs (RMSSD)**	Parasympathetic (vagal) activity	Time	Increase
**The standard deviation of IBIs (SDNN)**	Global HRV	Time	Increase
**High frequency power (HF, 0.15–0.4 Hz)**	Parasympathetic (vagal) activity	Frequency	Increase
**Total power**	Global HRV activity	Frequency	Increase

### Ethics

The study was conducted in accordance with the Helsinki declaration [51]. The Ethical Review Board (Stockholm) approved this study: 2018/2137–31. All participants signed a written informed consent form.

Ethical considerations informed the decision to use a two-week intervention period, as four treatments were not considered burdensome if no improvement was observed.

### Statistical analysis

#### Missing data

For NRS-11, Last Observation Carried Forward (LOCF) was used. HRV measurements only had a total of 11.8% missing data due to dropouts, missed appointments and cleaning of data. As the longitudinal modelling strategy used every available datapoint in an efficient way, it was not considered necessary to impute data.

#### Changes in pain and the association with changes in heart rate variability

Initially, participants were categorized based on the two strategies 1) MCID pain improvement and 2) Pain trajectories, as described above. To explore associations between changes in pain (both strategies) and changes in HRV, a series of linear mixed regression models was employed, using each HRV index as the outcome, including a person specific random intercept. The models included the pain groups (improved vs not improved or trajectories), time (baseline, 1 week, and follow-up), and the interaction between pain group and time. The estimate (β) of the interaction effect is interpreted as the difference between groups in the change in HRV (i.e. slope) between each time point. A significant interaction would indicate that the pain groups differ in the change in HRV over time.

An analysis adjusted for age, sex, baseline pain, and type of intervention was performed as a sensitivity analysis.

We estimated β with 95% CIs and *p*-values < 0.05 were considered statistically significant. Due to multiple testing, adjusting the significance level was considered. As power is already low in a secondary analysis, such adjustments would further reduce power and were considered not to benefit the understanding of the results and not to be critical in an exploratory analysis like this [52].

Continuous variables were reported as means with standard deviations and categorical variables as counts and percentages.

Group differences were graphically presented using box plots.

The analysis was performed using SPSS 27 [53] and Stata version 15 (StataCorp. 2017). The trajectory models were estimated with Stata package traj [34].

## Results

### Participants

Overall, the study group consisted of slightly more female than male participants with a mean age of 56 years, and most had suffered pain for several years. Most of the participants were living in a partnership. Most also experienced pain in other regions of the body. In general, they had experienced good effect from chiropractic treatment in the past and the mean score of the expectation to improve (0–10) was 5.9. Six-point seven percent of the data were excluded due to measurement errors based on visual inspection.

#### MCID pain improvement

Data from 88 patients were used as those with a non-clinically significant improvement (1-point improvement) (*n* = 39) were excluded from this analysis. The two pain categories (clinically improved vs not improved) differed in baseline pain intensity (NRS-11), with a mean of 5.5 (SD = 1.7) for the "improved" category and of 3.6 (SD = 2.3) for the "not improved" category. There were no differences in mean age and sex distribution between the categories (*p* > 0.05). The "improved" category (mean change in pain intensity -3.2 (SD 1.1)) also showed improvement in the disability (NDI) and qualitative characteristics of pain (McGill Questionnaire) compared to the "not improved" category. Further, the "improved" category reported less sick leave; two participants (5%) had been on sick leave due to NP the previous year, compared to six (14%) of the "not improved" category. Also, a slightly larger proportion of participants were classified in the medium or high STarT Back risk group (indicating a higher risk for future physical disability) in the "not-improved" category [39]. A higher number of participants with mid-back or low back pain was reported among the individuals in this category compared to the "improved" as seen in Table 2. The baseline differences in HRV between categories are presented in Supplementary File 1, where no significant baseline differences were observed.

**Table 2:** Demographic description of the study population at baseline divided into categories based on the clinically relevant change in pain intensity

	Improved (*n* = 44; 21 stretch, 23 stretch + SMT)	Not improved (*n* = 44; 18 stretch, 26 stretch + SMT)
Age, mean (sd)	56 (14.9)	60 (12.5)
Female, n (%)	24 (55)	26 (59)
Baseline pain intensity (NRS-11), mean (sd)	5.5 (1.7)	3.6 (2.3)
Pain duration		
More than 6 months, n (%)	6 (14)	8 (18)
Several years, n (%)	37 (84)	36 (82)
Pain intensity (NRS-11) change score, mean (sd)	-3.20 (1.1) *n* = 43	1.05 (1.2) *n* = 44
Neck disability change score, mean (sd)	-4.19 (4.5) *n* = 36	-1.31 (4.6) *n* = 36
STarT Back categories (Low risk: 0–3. Medium risk: Min. 4 points, max. 3 items from the psychosocial subscale. High risk: 4–5 on the psychosocial subscale.)		
Low risk, n (%)	34 (77)	31 (74)
Medium risk, n (%)	5 (11)	7 (17)
High risk, n (%)	2 (5)	4 (10)
Qualitative characteristics of pain (McGill questionnaire) change score, mean (sd)	-4.48 (5.5) *n* = 42	0.67 (8.4) *n* = 42
Type of occupation		
No job, n (%)	13 (30)	14 (32)
Mostly hard labour, n: (%)	1 (2)	1 (2)
Mostly a variation between hard and easy labour, n (%)	4 (9)	3 (7)
Mostly standing and walking, n (%)	9 (21)	9 (21)
Mostly sitting, n (%)	17 (39)	17 (39)
Quality of life (EQ-5D) change score, mean (sd)	0.007 (0.04) *n* = 30	-0.012 (0.08) *n* = 42
Arm pain, n (%)	27 (61)	25 (58)
Pain in the mid-back, n (%)	21 (48)	30 (68)
Pain in the low back, n (%)	23 (52)	28 (64)
Sick leave the previous year		
Do not work, n (%)	11 (25)	9 (26)
No sick leave, n (%)	31 (71)	29 (66)
Yes, between 1–7 days, n (%)	2 (5)	3 (7)
Yes, between 8–14 days, n (%)	0 (0)	1 (2)
Yes, more than 15 days, n (%)	0 (0)	2 (5)

**Fig. 2:**
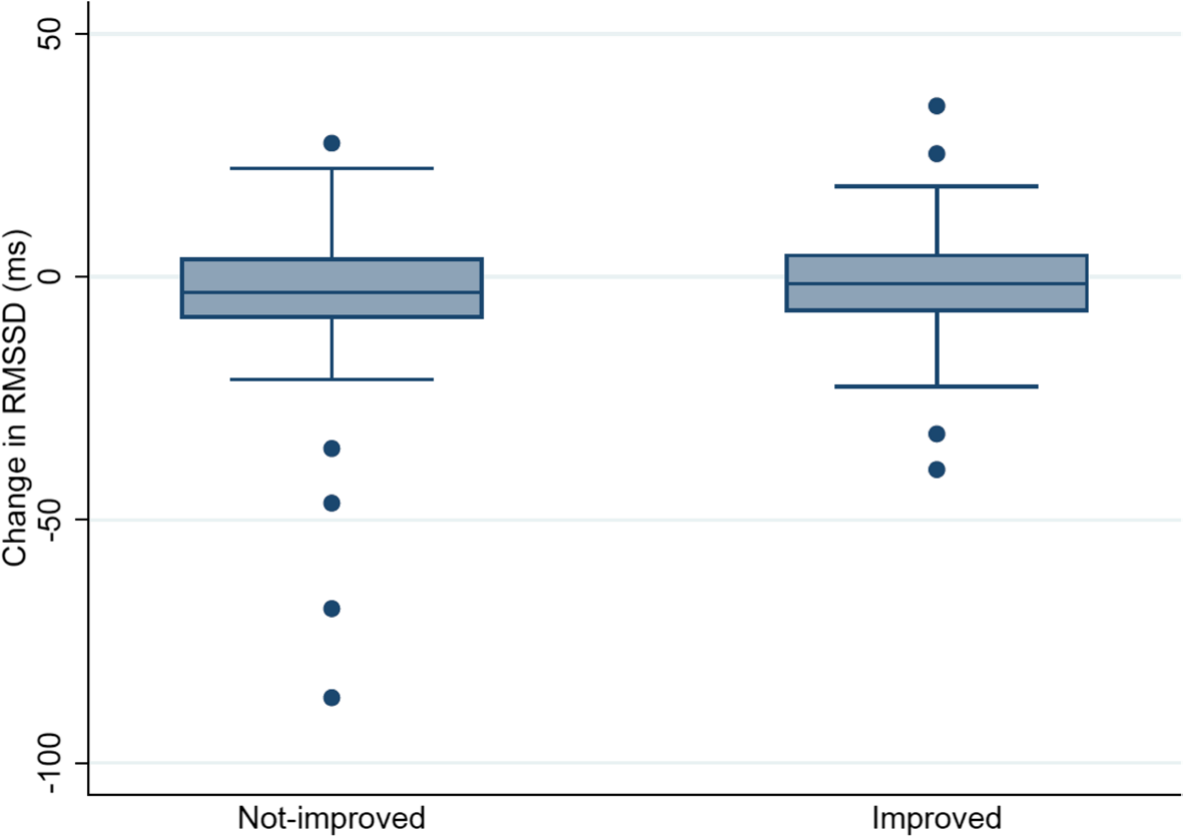
Mean change in RMSSD in the improved and not-improved categories

The mean change for RMSSD in both categories is presented in (Fig. 2). The HRV indices are presented in Supplementary files 2, 3, 4 and 5.

For the main HRV outcome RMSSD, a β-coefficient of the group-time interaction of 1.8 (CI = -2.2–5.7, *p*-value = 0.37) with the "not improved" category as the reference category was seen, meaning that the "improved" category showed an increased RMSSD of 1.8 units for each time point compared with the "not improved" category. All HRV indices are in favour of the "improved" category with small effect sizes, but estimates were not significant.

No significant changes between "improved" and "not-improved" categories were found for any of the HRV indices as seen in Table 3.

**Table 3:** Association between improvement categories (based on the clinically relevant change in pain intensity) and changes in HRV, using "no change" as the reference category (*n* = 88)

	β	*P*-value	Confidence intervals	
RR GroupxTime	0.5	0.96	-19.8	20.7
RMSSD GroupxTime	1.8	0.37	-2.2	5.7
SDNN GroupxTime	1.2	0.44	-1.9	4.4
HFms GroupxTime	59.7	0.18	-27.5	147.0
Total Power GroupxTime	51.7	0.56	-120.8	224.1

Adjustments for age, sex, baseline pain, and intervention did not affect our estimates. These results can be found in Supplementary File 6.

#### Pain trajectories

All participants from the RCT were included in the analysis (*n* = 127). As can be seen in (Fig. 1), a relatively stable pain course for all trajectories was observed from baseline to 14 days; the difference was mainly in pain intensity. Thus, there were no trajectories with a clear improvement or deterioration in pain over time. Overall, trajectory 1 had low levels of pain throughout the study, in contrast with trajectory 4, which had high levels of pain throughout. Trajectory 4 had a slight worsening in pain intensity over two weeks, while the other trajectories improved somewhat. The differences in the demographics of the trajectories are described in Table 4.

**Table 4:** Demographic description of the trajectories at baseline (n:127)

	Trajectory 1 (*n* = 29; 15 stretch, 14 stretch + SMT)	Trajectory 2 (*n* = 46; 26 stretch, 20 stretch + SMT)	Trajectory 3 (*n* = 40; 14 stretch, 26 stretch + SMT)	Trajectory 4 (*n* = 12; 6 stretch, 6 stretch + SMT)
Age, mean (sd)	54 (13.5)	60 (14.7)	58 (12.4)	54 (14.4)
Female, n (%)	13 (45)	25 (54)	24 (60)	9 (75)
Baseline pain intensity (NRS-11), mean (sd)	2.6 (1.3)	4.4 (1.9)	5.0 (1.8)	7.3 (1.0)
Pain duration				
Less than 6 months, n (%)	0 (0)	1 (2)	0 (0)	0 (0)
More than 6 months, n (%)	7 (24)	5 (11)	6 (15)	0 (0)
Several years, n (%)	22 (76)	37 (80)	34 (85)	12 (100)
Pain intensity (NRS-11) change score, mean (sd)	-1.46 (1.5) *n* = 28	-1.67 (2.2) *n* = 43	-0.39 (2.2) *n* = 36	0.08 (1.8) *n* = 12
Neck disability change score, mean (sd)	-3.9 (3.6) *n* = 22	-2.4 (4.7) *n* = 37	-1.7 (4.2) *n* = 36	-1.7 (5.0) *n* = 10
STarT Back categories (Low risk: 0–3. Medium risk: Min. 4 points, max. 3 items from the psychosocial subscale. High risk: 4–5 on the psychosocial subscale.)				
Low risk, n (%)	26 (90)	39 (91)	25 (63)	3 (25)
Medium risk, n (%)	1 (4)	2 (4)	9 (23)	6 (50)
High risk, n (%)	0 (0)	2 (4)	2 (5)	2 (17)
Qualitative characteristics of pain (McGill questionnaire) change score, mean (sd)	-2.3 (4.5) *n* = 26	-2.1 (5.9) *n* = 45	-0.9 (7.8) *n* = 39	3.3 (11.1) *n* = 12
Type of occupation				
Unemployed, n (%)	6 (21)	17 (37)	9 (23)	7 (58)
Mostly hard labour, n (%)	0 (0)	1 (2)	2 (5)	0 (0)
Mostly a variation between hard and easy labour, n (%)	1 (3)	4 (9)	4 (10)	0 (0)
4. Mostly standing and walking, n (%)	3 (10)	8 (17)	9 (23)	0 (0)
5. Mostly sitting, n (%)	19 (66)	17 (36)	15 (39)	5 (42)
EQ-5D change score, mean (sd)	-0.003 (0.072) *n* = 27	-0.005 (0.038) *n* = 43	0.007 (0.05) *n* = 36	-0.013 (0.11) *n* = 12
Arm pain, n (%)	10 (35)	27 (59)	28 (70)	10 (83)
Pain in the mid-back, n (%)	11 (40)	27 (59)	27 (68)	8 (67)
Pain in the lower back, n (%)	9 (31)	28 (61)	27 (68)	10 (83)
Sick leave the previous year				
Do not work, n (%)	3 (10)	17 (37)	7 (18)	4 (33)
No, n (%)	24 (83)	27 (59)	29 (73)	4 (33)
Yes, between 1–7 days, n (%)	1 (3)	1 (2)	2 (5)	1 (8)
Yes, between 8–14 days, n (%)	1 (3)	0 (0)	1 (3)	1 (8)
Yes, more than 15 days, n (%)	0 (0)	1 (2)	1 (3)	2 (17)

In short, there were more females in trajectory 4 (75% compared to 48–59% in the other trajectories) and these individuals were generally "worse", as 73% of the individuals in trajectory 4 had a STarT Back risk of medium or high (compared to the second-highest STarT Back risk group (trajectory 3) with 28% of individuals classified as medium or high risk), and half of the patients in trajectory 4 had been on sick leave the previous year (compared to below 11% of patients in all other trajectories).

Patients in trajectory 1 had a lower prevalence of pain in the arms, mid-back, or lower back compared to patients in the other trajectories and improved 3.9 points in the NDI, compared to the patients in the other trajectories who also improved, but only by 1.7 to 2.4.

In terms of the intervention and control groups in the RCT, patients were not evenly distributed among the trajectories. However, previous analyses on the same data set showed no significant differences between these groups for HRV and pain [27, 29]. No significant baseline differences in HRV between the trajectory groups were observed, (Supplementary File 7).

**Fig. 3:**
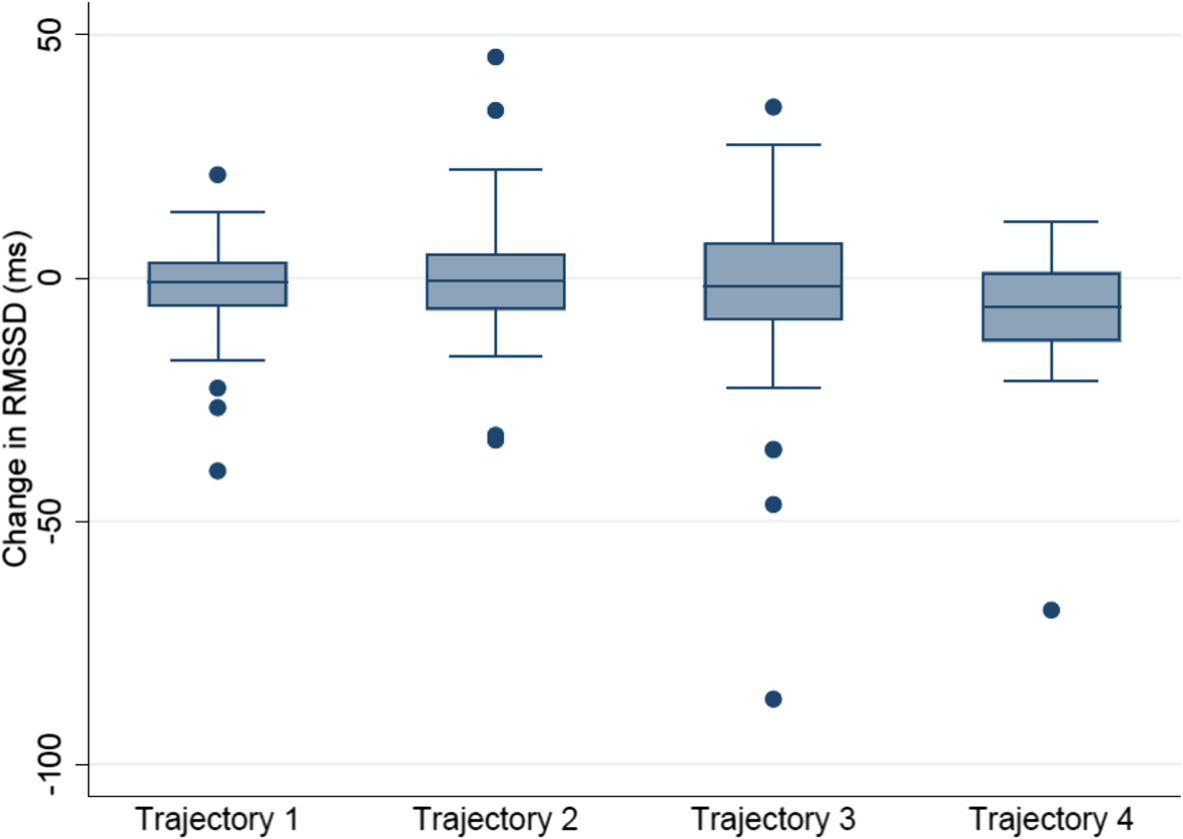
Mean change in RMSSD for the four pain trajectories

The mean change for RMSSD in all trajectories is presented in (Fig. 3). The rest of the HRV indices are presented in Supplementary Files 8, 9, 10 and 11.

The main HRV outcome, RMSSD, was not significantly different between trajectories 2–4 and 1, but a somewhat larger decrease in RMSSD over time was seen in trajectory 4, demonstrated by a β-coefficient of the group-time interaction of -3.8 (CI = -10.4 – 4.3, *p* = 0.26). The same non-significant estimates with a more substantial reduction on HRV with higher pain intensity was found for all HRV indices. The results are presented in Table 5 (linear mixed models).

**Table 5:** Association between pain trajectories (data-driven analysis) and changes in HRV over two weeks using trajectory 1 as the reference category (*n* = 127)

	Trajectory	β	*P*-value	Confidence intervals	
RR GroupxTime	2	14.9	0.19	-7.5	37.6
	3	10.3	0.38	-12.8	33.3
	4	-21.3	0.22	-55.1	12.5
RMSSD GroupxTime	2	3.0	0.42	-1.9	7.9
	3	-0.1	0.97	-4.9	5.1
	4	-3.8	0.26	-10.4	4.3
SDNN GroupxTime	2	1.4	0.44	-2.2	5.0
	3	-0.06	0.98	-3.7	3.6
	4	-3.0	0.28	-8.3	2.4
HFms GroupxTime	2	28.1	0.63	-86.6	142.8
	3	-43.7	0.47	-161.5	74.0
	4	-108.4	0.22	-281.1	64.1
Total Power GroupxTime	2	146.1	0.24	-97.3	389.4
	3	47.9	0.71	-202.0	297.7
	4	-130.0	0.49	-496.3	232.4

Adjusting for age, sex, baseline pain, and intervention did not significantly affect the estimates (Supplementary File 12).

## Discussion

The current study is an exploratory analysis of data from a RCT in which data from the randomization groups have been pooled, as no significant between-group differences were observed. We hypothesized that MCID in pain intensity would be associated with changes in HRV, but only small and statistically insignificant differences in HRV change were observed between patients with/without clinical improvement in pain intensity (NRS-11). Small and not statistically significant differences were also observed for HRV indices in individuals with different pain trajectories. However, due to the stability of the observed trajectories, the hypothesis that trajectories with consistent/fast improvement would show a beneficial increase in HRV compared to trajectories that were stable, could not be rejected or confirmed.

The study investigated patients with persistent or recurrent NP, a typically fluctuating condition [54–57]. The changes in pain during the course of treatment, which were the basis for the dichotomization into "improved" and "not improved", could have been due to normal fluctuations in pain and not a reflection of important clinical improvements as intended. We sought to minimize this random fluctuation by ignoring minor pain changes. Further, there was a difference in baseline pain between individuals who improved compared to those who did not. The base for the categorization of "improved" and "not improved" made it more likely for the patients with high initial pain scores to reach a pain reduction of 2/10 or more (flooring effect). Possibly, more patients would have reached this level if an inclusion criterion in the study was pain intensity of a certain (higher) level at baseline. Also, overall higher pain levels in this category may have had a stronger impact on HRV already at baseline. However, adjusting for baseline differences in pain intensity did not change the conclusion of this study.

Classifying different trajectories of pain during an intervention period is a way of investigating pain development during a given time period, used in previous studies in patients with NP in a clinical setting [57]. The identified pain trajectories had very different baseline pain intensities. This reflects the inclusion of participants with varying pain levels, as expected in the condition under investigation. However, the trajectories were all stable over time, which could not have been foreseen. The non-significant deterioration in HRV found in the trajectory with high pain intensity (trajectory 4) can be due to a relationship between persistent pain and HRV, possibly causing patients classified in this trajectory to be less susceptible to improvement. Certainly, the patients in this trajectory had several other known factors associated with poor outcomes.

It is possible that four treatments over two weeks were not enough to obtain pain relief sufficient to influence HRV in patients with chronic pain. The intervention period was chosen based on previous research on HRV and SMT which focuses on the immediate effects of manual therapy on HRV [24, 25, 58–61]. Four treatments over two weeks was considered sufficient to detect a change in pain based on previous research, which found that improvement after four treatments predicts improvement in persistent low back pain after three and twelve months [62]. In the study by Leboeuf-Yde et al. [62], it was also noted that a lack of an early treatment response was not remedied during the rest of the treatment programme. By delivering four treatments for persistent or recurrent NP, we therefore expected to identify responders to care. It is possible that NP sufferers differ from the persistent low back pain sufferers in response to treatment, but this is not supported by the literature [63]. It is even observable that low back pain sufferers are equally or more affected with regards to emotional situation and disability level, and often have longer pain duration than NP sufferers [64]. As reported in Table 2, most individuals also experienced pain in other body regions, and a lack of association between changes in NP and HRV may be partly explained by concomitant pain.

Treatment content was collected for, and all treatment modalities were in line with what has been reported in other studies [65–67]. The stretching exercises used were described in a previous study [11], but also found to be commonly used. The photographs used to illustrate the exercises were found on a Swedish chiropractic website (and used with permission). Thus, we believe that the interventions used are relevant for manual professions.

The results did not appear to support the proposed mechanism that changes in pain lead to changes in ANS. It is possible that any changes in HRV following pain relief have a latency extending beyond the two weeks observed in the current study, which is supported by previous research where a reduction of NP has been observed when patients received treatment aimed at improving HRV over ten weeks [23]. It is also possible that any mechanistic interrelatedness of pain and ANS balance is simply obscured by the multitude of other factors affecting one, the other, or both. Further, there were several limitations related to HRV measurement that may have influenced results, such as lack of 24-h assessment of HRV and few measurements over time, which may have yielded more stable measures of resting HRV.

The reliability of the 5-min HRV measurement of parasympathetic indices at rest, is, however, considered good [44].

Different strategies were used to reduce the risk of selection and attention bias, as described in Additional file 1: Appendix A and previously published articles [27, 29]. Any inter-relatedness of NP and HRV is possibly more complicated and confounding factors may exist which we are not aware of. The patients' internal factors such as emotions, unknown underlying diseases and stress influencing HRV [4, 68–70] could not be controlled for. Participation in the study may have led to more daily stress as time had to be set aside to participate. The initial study design also included a conditioned pain modulation test, where the hand was submerged in cold water. This was done after the HRV measurements to avoid any effect of the test on HRV, but it is possible that the anticipation of the uncomfortable test influenced HRV. The effect of these confounding factors would potentially reduce the observed association between reduction in pain and change in HRV over two weeks. This was addressed by applying a strict measuring protocol.

### Methodological considerations

The measurement procedures were standardized and well-controlled, with two trained researchers performing all HRV measurements. The study utilized repeated measures of all outcomes. Two different strategies to classify pain over two weeks were used in order to investigate this relationship in a thorough manner. This is the first study of its kind investigating the relationship between changes in pain and changes in HRV during a two-week treatment intervention for this population.

As this is an exploratory analysis based on data from a trial with a power calculation for logarithmic values with larger group sizes [27], the analysis undertaken with smaller group sizes was underpowered. Therefore, caution is warranted when interpreting the estimates.

HRV is prone to measurement error. About 40% of the variance in HRV is known to be explained by situational effects and person-situation interaction [44]. It is also acknowledged that the HRV measurements are prone to be affected by factors such as psychological distress, training status, and time of the day the day measurements are taken, which are not possible to control for. HRV varies from day to day for each individual as well as between individuals, one measure each day at three occasions may have rendered uncertain results and may have reduced power further. [71, 72]. Considering the original RCT design of the study, measuring each patient’s individual HRV values over time, before the intervention period commenced, was not possible. If this had been possible, investigating whether the patients HRV changes from their “normal” would yield stronger certainty in the results.

### Generalizability

Participants were mainly excluded from the main trial due to factors, conditions or medications known to influence HRV-measurements. These included medications such as antidepressants. Thus, the study participants may have been physically and psychologically healthy compared to the general population with persistent or recurrent NP. However, there is evidence that patients with pain and depression undergoing medical treatment for depression experience reduced pain and improved daily function [73, 74]. Hence, the excluded patients may not have differed in their pain response from other persistent or recurrent NP sufferers. The results in this study may be considered generalizable for chronic pain conditions based on previous research investigating chronic pain and HRV [1–3], even though strong conclusions cannot be drawn from the results in this study as the observed associations are weak and uncertain.

## Conclusion

Changes in pain intensity during an intervention with SMT and/or home stretching exercises over two weeks was not significantly associated with changes in HRV for this study population with persistent or recurrent NP. The results do not favour the link between pain and HRV in this patient group. Future studies should rely on more frequent measurements of HRV during longer treatment periods.

## Supplementary Information

**Additional file 1: Appendix A.** Details about the RCT.

**Additional file 2: Appendix B.** Stretch exercises to perform daily for 14 days.

**Additional file 3: Supplementary file 1. **Association between pain groups (based on clinically relevant change in pain intensity) and differences in HRV at baseline, using "no change" as the reference category) (*n*=88).

**Additional file 4: Supplementary file 2. **Mean change in R-R in the improved and not-improved categories.

**Additional file 5: Supplementary file 3.** Mean change in SDNN in the improved and not-improved categories.

**Additional file 6: Supplementary file 4. **Mean change in HF in the improved and not-improved categories.

**Additional file 7: Supplementary file 5. **Mean change in Total Power in the improved and not-improved categories.

**Additional file 8: Supplementary file 6.** Association between pain groups (based on clinically relevant change in pain intensity) and changes in HRV at each time point, using "no change" as the reference category) (*n*=87), adjusted for age, sex, baseline pain and intervention.

**Additional file 9: Supplementary file 7.** Association between pain trajectories and HRV at baseline, using group 1. as the reference category (*n*=125).

**Additional file 10: Supplementary file 8.** Mean change in R-R intervals for the four pain trajectories.

**Additional file 11: Supplementary file 9.** Mean change in SDNN for the four pain trajectories.

**Additional file 12: Supplementary file 10.** Mean change in HF for the four pain trajectories.

**Additional file 13: Supplementary file 11.** Mean change in Total Power for the four pain trajectories.

**Additional file 14: Supplementary file 12. **Association between pain trajectories and changes in HRV, using group 1. as the reference category (*n*=125), adjusted for age, sex, baseline pain and intervention.

## Data Availability

The data that support the findings of this study were used under license for the current study. Restrictions apply to the availability of these data, hence they are not publicly available. Data can, however, be obtained from the authors upon reasonable request to the main author (AGB), and with permission of Karolinska Institutet.

## Abbreviations

AIC: Akaike Information Criteria
ANS: Autonomic Nervous System
ECG: Electrocardiography
HRV: Heart Rate Variability
ICF: International Classification of Functioning, Disability, and Health
LOCF: Last Observation Carried Forward
MCID: Minimal Clinical Important Difference
NDI: Neck Disability Index
NP: Neck Pain
NRS-11: 11-Point Numeric Rating Scale
RMSSD: Root Mean Squared Successive Differences
SMT: Spinal Manipulative Therapy
