# Supplementary material for: Are changes in pain associated with changes in heart rate variability in patients treated for recurrent or persistent neck pain?
Source: BMC Musculoskelet Disord. 2022 Oct 4;23:895. doi: 10.1186/s12891-022-05842-4 (PMC9531383; doi:10.1186/s12891-022-05842-4)
Supplement: Supplementary file 1 — Additional file 1: Appendix A. Details about the RCT. [file 12891_2022_5842_MOESM1_ESM.docx]

Appendix A: Details about the RCT.

RCT setting

The study was conducted at five clinics that were part of the Stockholm regional health service. They were all multi-professional, employing chiropractors, dieticians, occupational therapists, and physiotherapists. The chiropractors who took part in the study were licensed by the Swedish National Board of Health and Welfare. They received written information and attended workshops prior to the study. The primary researcher (AGB) screened potential participants for eligibility. Eligible participants were enrolled in the study and booked for a series of five treatments over two weeks.

Three hundred thirteen potential participants were screened for eligibility, and 156 were consequently excluded due to various exclusion criteria. One hundred fifty-seven patients were included; 26 out of these could not participate when the data collection commenced.

Baseline

The patients received participant information and a consent form to be signed before inclusion in the study. The baseline visit commenced with the patients answering various questions relating to demographics, pain, and disability. Then a measurement of HRV was undertaken. Directly after this procedure, the first study visit commenced.

Intervention

The patients received either home stretching exercises and SMT or home stretching exercises only. The treating chiropractor adapted the application of SMT to each patient based on their clinical findings, applying high-velocity low amplitude thrusts or mobilization to any spinal joints within the limitations defined in the study protocol (31).

Manual techniques were combined in most of the SMT treatment sessions. The techniques were, in decreasing order: Diversified technique (80% of all subjects received this modality), mobilization (48% of subjects), activator (spring-loaded instrument) (42% of subjects), as well as other modalities (2%-31%). The stretching exercises were performed at least 10 out of 14 days of all subjects as previously reported (30).

The home stretching exercises used in this study were developed by Ylinen et al. (10) and consisted of four different stretches that took approximately 10 minutes to perform (Please see Appendix B). All patients were asked to keep an exercise diary to monitor adherence to the exercises.

All patients were scheduled for five visits to the clinic and underwent the same physical examination at each visit. Two weeks and four visits were chosen based on previous research on the effect of manual therapy on HRV and pain (15, 17, 25, 33-35) and was considered sufficient to detect a clinically relevant change in pain intensity between groups.

Bias

Selection bias could have occurred on the basis of subjects seeking care at the clinics where the study was undertaken if the patients had a previous positive experience with the allocated chiropractor. This was addressed by choosing multimodal clinics, advertising in local newspapers, and asking local general practitioners' offices to inform their patients about the study.

As the chiropractors performing the treatments could not be blinded, potential bias favouring the intervention group was addressed by providing verbal and written information on how to interact with the subjects. All patients also had to undergo a physical examination on each visit to ensure that both groups experienced physical contact.

Interventions and measurements

The researchers arranged workshops and observed each other when performing the measurements to assure similarity of the procedures. The chiropractors who participated in the study were well informed (written information and a workshop) about the study procedures and were experienced in treating this patient group. Further, they were instructed and guided to provide as-similar-as-possible clinical encounters for all subjects.

Power

The sample size for the RCT was calculated a priori. This was based on the primary outcome of HRV, RMSSD (47), described in table 1. The calculations were based on values obtained from the study by Hallman et al. (24). They found that to reach a power of 80%, with a significance level of 5%, sixty subjects were needed to detect a change of 10% between groups (48).

Generalizability

125 out of 313 screened participants (40%) were included. Possible participants were excluded from the study based on the inclusion/exclusion criteria requirements for HRV measurements.
