# Supplementary material for: Are changes in pain associated with changes in heart rate variability in patients treated for recurrent or persistent neck pain?
Source: BMC Musculoskelet Disord. 2022 Oct 4;23:895. doi: 10.1186/s12891-022-05842-4 (PMC9531383; doi:10.1186/s12891-022-05842-4)
Supplement: Supplementary file 2 — Additional file 2: Appendix B. Stretch exercises to perform daily for 14 days. [file 12891_2022_5842_MOESM2_ESM.docx]

Appendix B.

**Stretch exercises to perform daily for 14 days**

*The exercises should be done every day for 14 days. It will take you approximately 10 minutes.*

*It is highly important that the exercises are done as described.*

*Use the exercise diary (last page) as a reminder to do your exercises.*

**Each exercise is done for 30 seconds and repeated 3 times.**

1. Bend your head to the left. Place your left arm over your head and feel the stretch.
2. Bend your head to the right. Place your right arm over your head and feel the stretch.


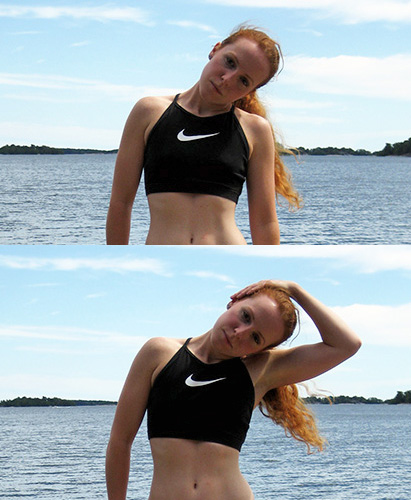


1. Bend and rotate the head to the left. Place your left arm over your head and feel the stretch.
2. Bend and rotate the head to the right. Place your right arm over your head and feel the stretch.


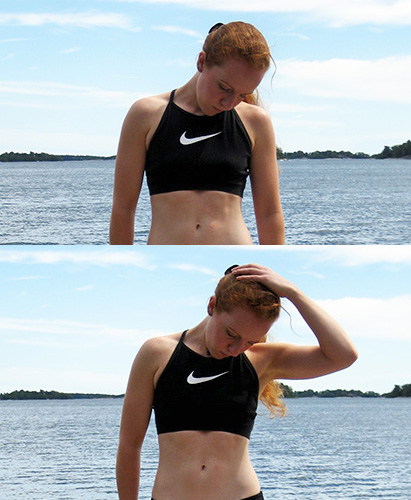


5: Bend your head forward. Put an arm over your head and feel the stretch.


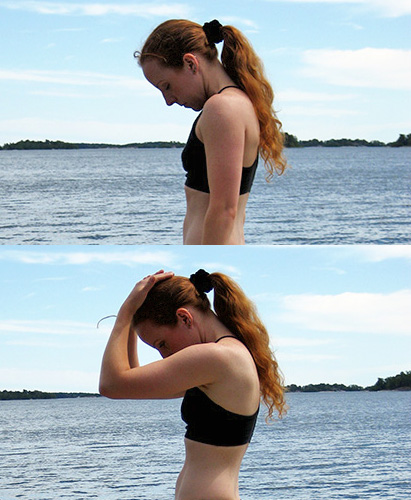


6: Finish by sitting up straight or lying flat on your back. Make a double chin by pulling the chin towards you and hold for 3-5 seconds. Do this 5 times.


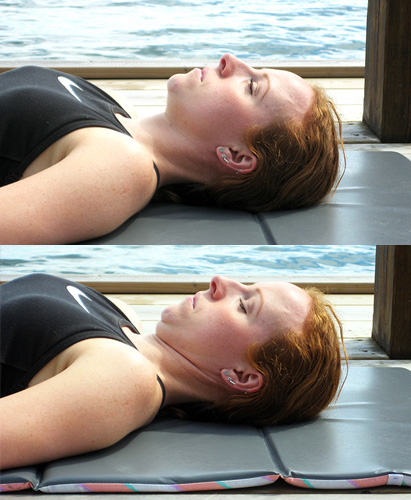


Good luck!

## Datum för konsultation hos kiropraktor :_______________

KOD: _____________

Ringa in ditt svar varje dag:

Dag 1: Stretching utfört enligt instruktion Ja Nej

Dag 2: Stretching utfört enligt instruktion Ja Nej

Dag 3: Stretching utfört enligt instruktion Ja Nej

Dag 4: Stretching utfört enligt instruktion Ja Nej

Dag 5: Stretching utfört enligt instruktion Ja Nej

Dag 6: Stretching utfört enligt instruktion Ja Nej

Dag 7: Stretching utfört enligt instruktion Ja Nej

Dag 8: Stretching utfört enligt instruktion Ja Nej

Dag 9: Stretching utfört enligt instruktion Ja Nej

Dag 10: Stretching utfört enligt instruktion Ja Nej

Dag 11: Stretching utfört enligt instruktion Ja Nej

Dag 12: Stretching utfört enligt instruktion Ja Nej

Dag 13: Stretching utfört enligt instruktion Ja Nej

Dag 14: Stretching utfört enligt instruktion Ja Nej

Eventuella kommentarer: _________________________________________________________

______________________________________________________________________________
