# Supplementary material for: Are changes in pain associated with changes in heart rate variability in patients treated for recurrent or persistent neck pain?
Source: BMC Musculoskelet Disord. 2022 Oct 4;23:895. doi: 10.1186/s12891-022-05842-4 (PMC9531383; doi:10.1186/s12891-022-05842-4)
Supplement: Supplementary file 3 — Additional file 3: Supplementary file 1. Association between pain groups (based on clinically relevant change in pain intensity) and differences in HRV at baseline, using "no change" as the reference category) (n=88). [file 12891_2022_5842_MOESM3_ESM.docx]

Supplementary file 1.

**Association between pain groups (based on clinically relevant change in pain intensity) and differences in HRV at baseline, using "no change" as the reference category) (n=88)**

|  | | |  |  |
| --- | --- | --- | --- | --- |
|  | β | P-value | Confidence intervals | |
| RR Baseline group difference | 3.2 | 0.93 | -65.7 | 72.1 |
| RMSSD Baseline group difference | -5.3 | 0.35 | -16.5 | 5.9 |
| SDNN Baseline group difference | -2.2 | 0.62 | -10.9 | 6.5 |
| HFms Baseline group difference | -158.9 | 0.16 | -383.1 | 65.2 |
| Total Power Baseline group difference | 9.1 | 0.97 | -482.4 | 500.6 |
