# Supplementary figures and images for: Are changes in pain associated with changes in heart rate variability in patients treated for recurrent or persistent neck pain?
Source: BMC Musculoskelet Disord. 2022 Oct 4;23:895. doi: 10.1186/s12891-022-05842-4 (PMC9531383; doi:10.1186/s12891-022-05842-4)

Supplementary file 2.

Mean change in R-R in the improved and not-improved categories.


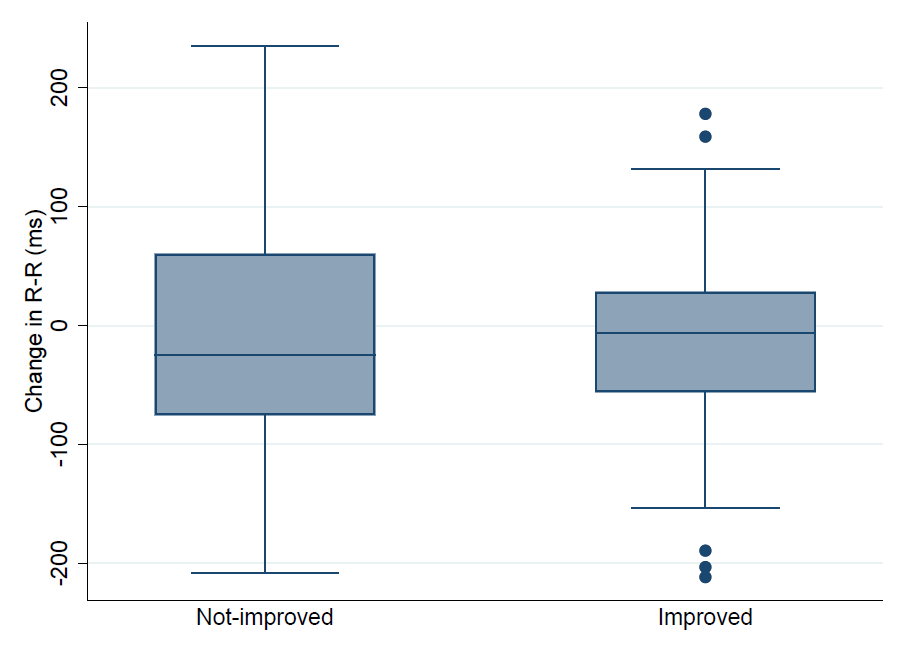

Supplement: Supplementary file 4 — Additional file 4: Supplementary file 2. Mean change in R-R in the improved and not-improved categories. [file 12891_2022_5842_MOESM4_ESM.docx]

Supplementary file 3.

Mean change in SDNN in the improved and not-improved categories.


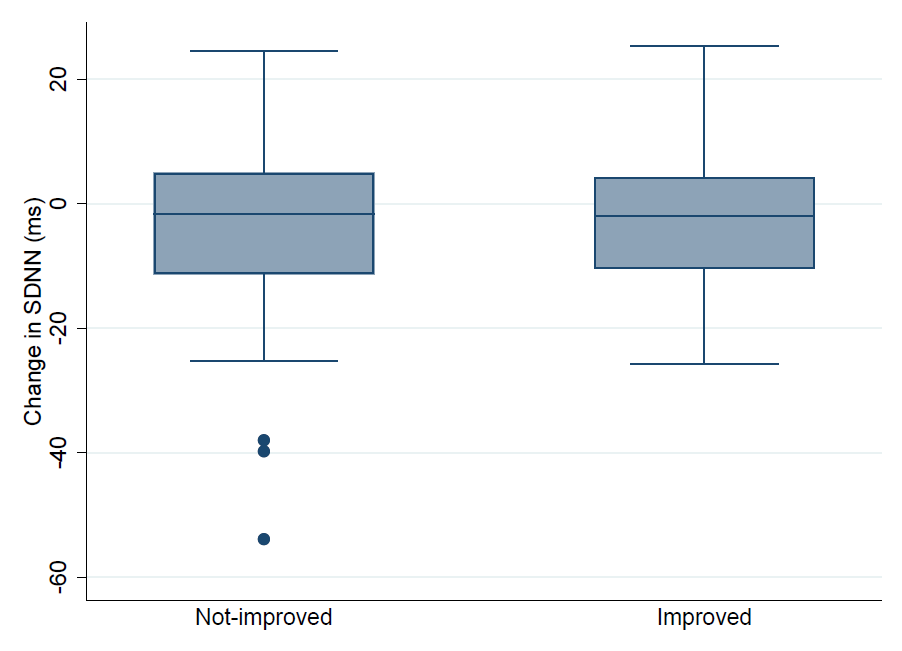

Supplement: Supplementary file 5 — Additional file 5: Supplementary file 3. Mean change in SDNN in the improved and not-improved categories. [file 12891_2022_5842_MOESM5_ESM.docx]

Supplementary file 4.

Mean change in HF in the improved and not-improved categories.


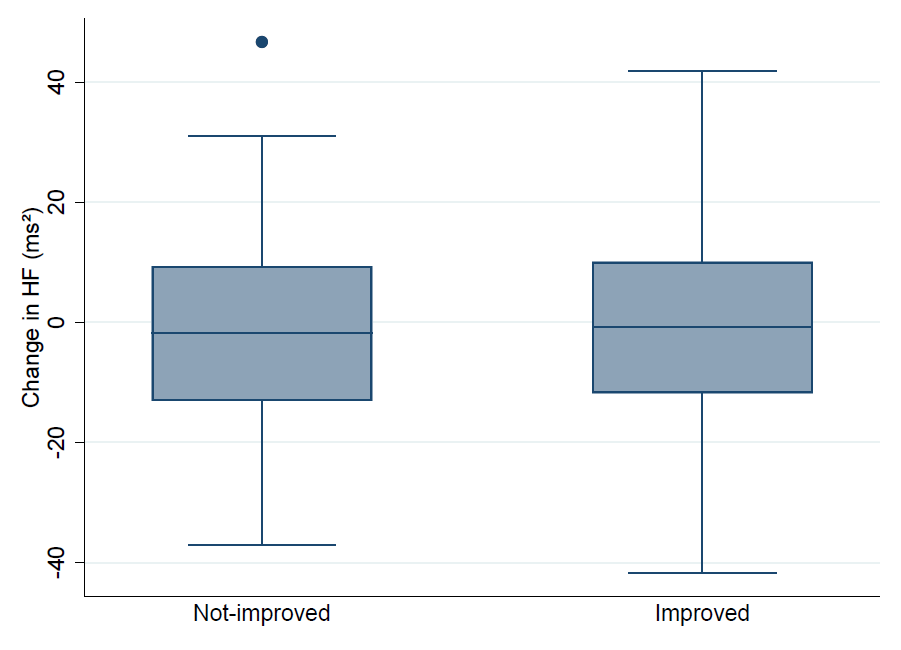

Supplement: Supplementary file 6 — Additional file 6: Supplementary file 4. Mean change in HF in the improved and not-improved categories. [file 12891_2022_5842_MOESM6_ESM.docx]

Supplementary file 5.

Mean change in Total Power in the improved and not-improved categories.


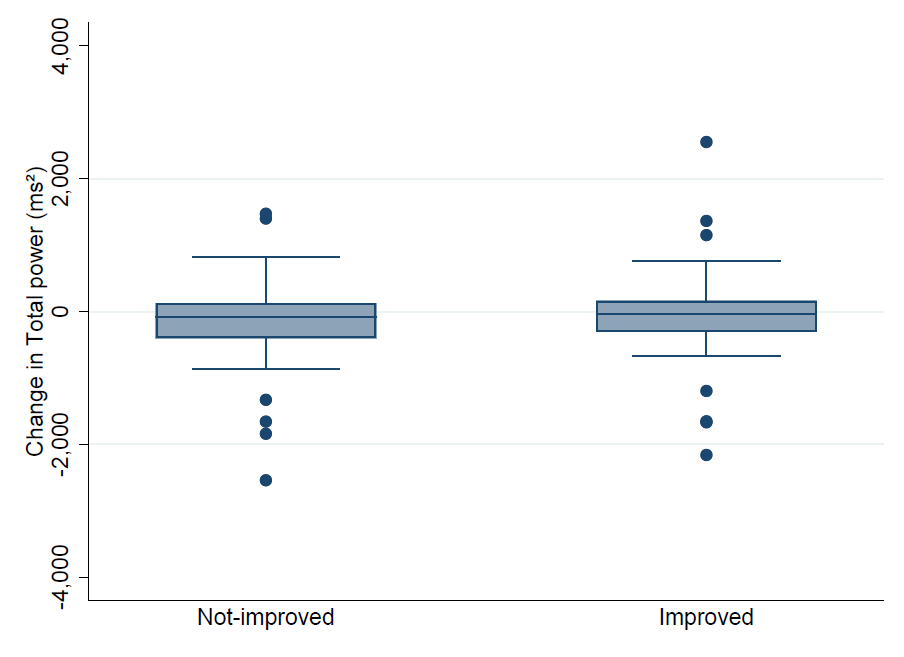

Supplement: Supplementary file 7 — Additional file 7: Supplementary file 5. Mean change in Total Power in the improved and not-improved categories. [file 12891_2022_5842_MOESM7_ESM.docx]

Supplementary file 8.

Mean change in R-R intervals for the four pain trajectories.


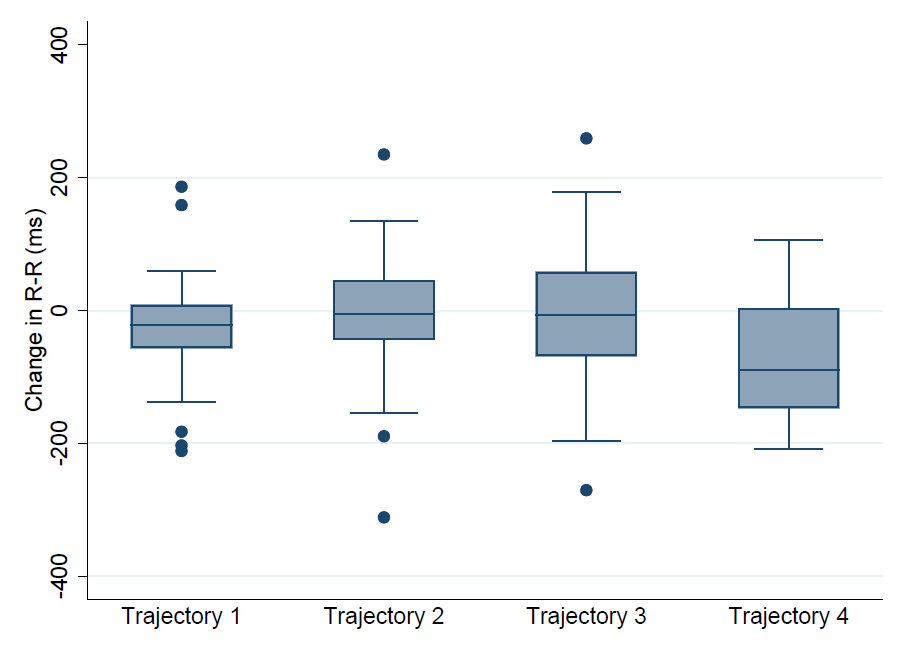

Supplement: Supplementary file 10 — Additional file 10: Supplementary file 8. Mean change in R-R intervals for the four pain trajectories. [file 12891_2022_5842_MOESM10_ESM.docx]

Supplementary file 9.

Mean change in SDNN for the four pain trajectories.


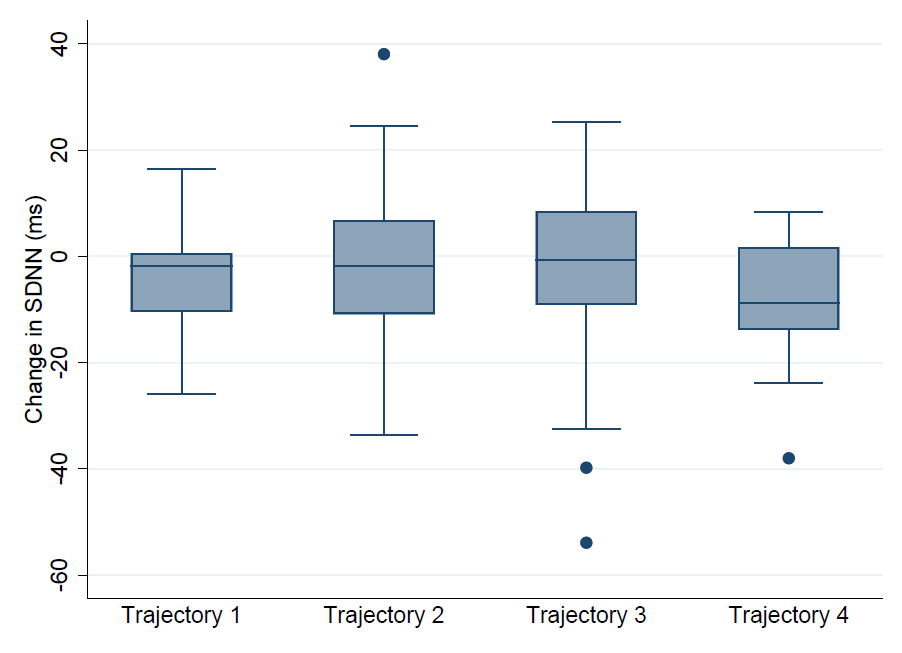

Supplement: Supplementary file 11 — Additional file 11: Supplementary file 9. Mean change in SDNN for the four pain trajectories. [file 12891_2022_5842_MOESM11_ESM.docx]

Supplementary file 10.

Mean change in HF for the four pain trajectories.


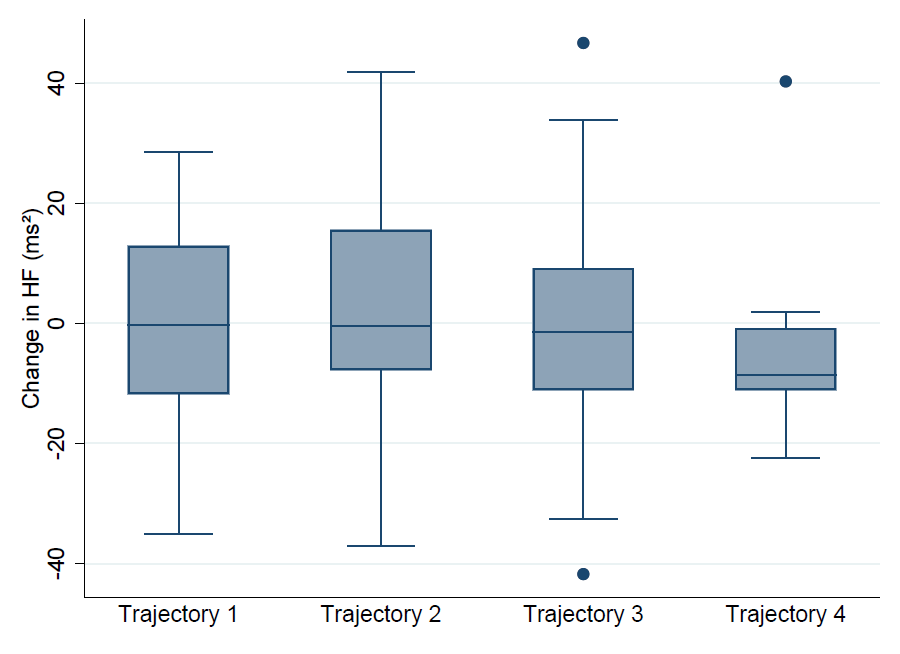

Supplement: Supplementary file 12 — Additional file 12: Supplementary file 10. Mean change in HF for the four pain trajectories. [file 12891_2022_5842_MOESM12_ESM.docx]

Supplementary file 11. Mean change in Total Power for the four pain trajectories.


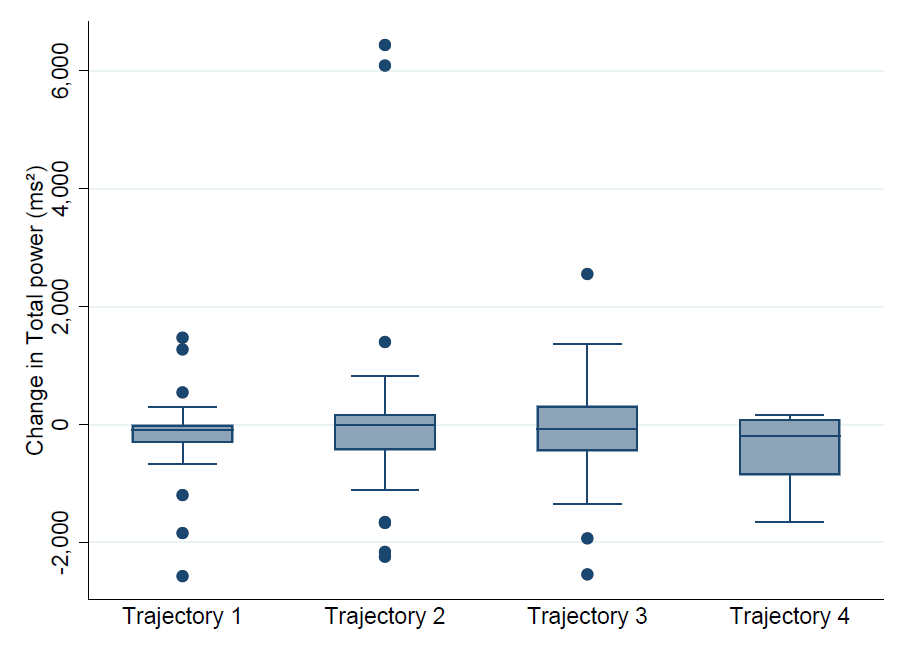

Supplement: Supplementary file 13 — Additional file 13: Supplementary file 11. Mean change in Total Power for the four pain trajectories. [file 12891_2022_5842_MOESM13_ESM.docx]
