# Supplementary material for: Are changes in pain associated with changes in heart rate variability in patients treated for recurrent or persistent neck pain?
Source: BMC Musculoskelet Disord. 2022 Oct 4;23:895. doi: 10.1186/s12891-022-05842-4 (PMC9531383; doi:10.1186/s12891-022-05842-4)
Supplement: Supplementary file 8 — Additional file 8: Supplementary file 6. Association between pain groups (based on clinically relevant change in pain intensity) and changes in HRV at each time point, using "no change" as the reference category) (n=87), adjusted for age, sex, baseline pain and intervention. [file 12891_2022_5842_MOESM8_ESM.docx]

Supplementary file 6.

**Association between pain groups (based on clinically relevant change in pain intensity) and changes in HRV at each time point, using "no change" as the reference category) (n=87), adjusted for age, sex, baseline pain and intervention.**

| Group x Time | β | P-value | Confidence intervals | |
| --- | --- | --- | --- | --- |
| R-R (ms) | -9.9 | 0.43 | -34.7 | 14.8 |
| RMSSD (ms) | 1.8 | 0.45 | -2.9 | 6.6 |
| SDNN (ms) | 0.8 | 0.69 | -3.0 | 4.5 |
| HF (ms^2^) | 49.7 | 0.34 | -53.5 | 152.9 |
| Total Power (ms^2^) | -18.9 | 0.86 | -228.1 | 190.3 |
