# Supplementary material for: Are changes in pain associated with changes in heart rate variability in patients treated for recurrent or persistent neck pain?
Source: BMC Musculoskelet Disord. 2022 Oct 4;23:895. doi: 10.1186/s12891-022-05842-4 (PMC9531383; doi:10.1186/s12891-022-05842-4)
Supplement: Supplementary file 9 — Additional file 9: Supplementary file 7. Association between pain trajectories and HRV at baseline, using group 1. as the reference category (n=125). [file 12891_2022_5842_MOESM9_ESM.docx]

Supplementary file 7.

**Association between pain trajectories and HRV at baseline, using group 1. as the reference category (n=125).**

|  | Group | β | P-value | Confidence intervals | |
| --- | --- | --- | --- | --- | --- |
| RR GroupxTime | 2 | -16.4 | 0.66 | -90.2 | 57.5 |
|  | 3 | -41.2 | 0.29 | -117.2 | 34.9 |
|  | 4 | -7.3 | 0.90 | -119.3 | 104.6 |
| RMSSD GroupxTime | 2 | -8.4 | 0.22 | -21.9 | 5.1 |
|  | 3 | 3.3 | 0.64 | -10.6 | 17.2 |
|  | 4 | 2.5 | 0.81 | -17.9 | 23.0 |
| SDNN GroupxTime | 2 | -7.2 | 0.18 | -17.2 | 2.8 |
|  | 3 | -0.7 | 0.89 | -11.0 | 9.6 |
|  | 4 | 0.4 | 0.96 | -14.8 | 15.6 |
| HFms GroupxTime | 2 | -116.7 | 0.43 | -409.0 | 175.6 |
|  | 3 | 101.5 | 0.51 | -199.1 | 402.0 |
|  | 4 | 174.1 | 0.44 | -267.7 | 615.9 |
| Total Power GroupxTime | 2 | -535.7 | 0.13 | -1232.2 | 160.8 |
|  | 3 | -306.7 | 0.40 | -1023.3 | 410.0 |
|  | 4 | -136.5 | 0.80 | -1190.9 | 918.0 |
