# Supplementary material for: Are changes in pain associated with changes in heart rate variability in patients treated for recurrent or persistent neck pain?
Source: BMC Musculoskelet Disord. 2022 Oct 4;23:895. doi: 10.1186/s12891-022-05842-4 (PMC9531383; doi:10.1186/s12891-022-05842-4)
Supplement: Supplementary file 14 — Additional file 14: Supplementary file 12. Association between pain trajectories and changes in HRV, using group 1. as the reference category (n=125), adjusted for age, sex, baseline pain and intervention. [file 12891_2022_5842_MOESM14_ESM.docx]

Supplementary file 12.

**Association between pain trajectories and changes in HRV, using group 1. as the reference category (n=125), adjusted for age, sex, baseline pain and intervention.**

|  | Group | β | P-value | Confidence intervals | |
| --- | --- | --- | --- | --- | --- |
| RR GroupxTime | 2 | 10.4 | 0.37 | -12.4 | 33.1 |
|  | 3 | 9.0 | 0.45 | -14.5 | 32.5 |
|  | 4 | -21.0 | 0.23 | -55.3 | 13.4 |
| RMSSD GroupxTime | 2 | 2.4 | 0.34 | -2.6 | 7.4 |
|  | 3 | -0.1 | 0.97 | -5.2 | 5.0 |
|  | 4 | -3.0 | 0.43 | -10.5 | 4.5 |
| SDNN GroupxTime | 2 | 0.90 | 0.63 | -2.7 | 4.5 |
|  | 3 | -0.16 | 0.93 | -3.9 | 3.6 |
|  | 4 | -2.8 | 0.32 | -8.3 | 2.7 |
| HFms GroupxTime | 2 | 18.4 | 0.76 | -99.0 | 135.6 |
|  | 3 | -41.9 | 0.50 | -163.3 | 79.5 |
|  | 4 | -98.7 | 0.27 | -276.2 | 78.8 |
| Total Power GroupxTime | 2 | 126.5 | 0.32 | -123.7 | 376.7 |
|  | 3 | 54.4 | 0.68 | -204.3 | 313.1 |
|  | 4 | -104.6 | 0.59 | -482.6 | 273.4 |
